# Network Modeling of Complex Time-Dependent Changes in Patient Adherence to Adjuvant Endocrine Treatment in ER+ Breast Cancer

**DOI:** 10.3389/fpsyg.2022.856813

**Published:** 2022-07-12

**Authors:** Eileen H. Shinn, Brooke E. Busch, Neda Jasemi, Cole A. Lyman, J. Tory Toole, Spencer C. Richman, William Fraser Symmans, Mariana Chavez-MacGregor, Susan K. Peterson, Gordon Broderick

**Affiliations:** ^1^Department of Behavioral Science, The University of Texas MD Anderson Cancer Center, Houston, TX, United States; ^2^Center for Clinical Systems Biology, Rochester General Hospital, Rochester, NY, United States; ^3^Department of Pathology, The University of Texas MD Anderson Cancer Center, Houston, TX, United States; ^4^Department of Health Services Research, The University of Texas MD Anderson Cancer Center, Houston, TX, United States; ^5^Department of Breast Medical Oncology, The University of Texas MD Anderson Cancer Center, Houston, TX, United States

**Keywords:** hormone receptor-positive breast cancer, adherence, behavioral feedback network, computational modeling, forecast prediction, rescue strategy

## Abstract

Early patient discontinuation from adjuvant endocrine treatment (ET) is multifactorial and complex: Patients must adapt to various challenges and make the best decisions they can within changing contexts over time. Predictive models are needed that can account for the changing influence of multiple factors over time as well as decisional uncertainty due to incomplete data. AtlasTi8 analyses of longitudinal interview data from 82 estrogen receptor-positive (ER+) breast cancer patients generated a model conceptualizing patient-, patient-provider relationship, and treatment-related influences on early discontinuation. Prospective self-report data from validated psychometric measures were discretized and constrained into a decisional logic network to refine and validate the conceptual model. Minimal intervention set (MIS) optimization identified parsimonious intervention strategies that reversed discontinuation paths back to adherence. Logic network simulation produced 96 candidate decisional models which accounted for 75% of the coordinated changes in the 16 network nodes over time. Collectively the models supported 15 persistent end-states, all discontinued. The 15 end-states were characterized by median levels of general anxiety and low levels of perceived recurrence risk, quality of life (QoL) and ET side effects. MIS optimization identified 3 effective interventions: reducing general anxiety, reinforcing pill-taking routines, and increasing trust in healthcare providers. Increasing health literacy also improved adherence for patients without a college degree. Given complex regulatory networks’ intractability to end-state identification, the predictive models performed reasonably well in identifying specific discontinuation profiles and potentially effective interventions.

## Introduction

Adjuvant endocrine therapy (ET) for patients with stage I-III hormone receptor-positive breast cancer reduces the rate of breast cancer recurrence by almost 50% during the first 5 years of ET (EBCTCG, 2005; Davies et al., 2011). Despite its benefit, roughly 50% (Hershman et al., 2010) of patients discontinue ET before completing their fifth year; 14–30% discontinue within the second year (Baum et al., 2002; Coombes et al., 2004). Large clinical trial and prospective cohort studies using medical record data from single-payor healthcare systems have found that age over 65 or younger than 45 (Hershman et al., 2010), race (non-white) (Wheeler et al., 2019), education level of high school degree or lower (Wulaningsih et al., 2018), co-pay amounts greater than $90/month (Neugut et al., 2011), lower disease stage (Wulaningsih et al., 2018), and multiple comorbidities requiring complex medication schedules are associated with non-adherence (Neugut et al., 2011; Sheppard et al., 2014). Other observational cohort studies which collected patient questionnaire data have cited ET side effects, forgetfulness, low perceived risk of recurrence (Fink et al., 2004), poor communication with healthcare provider (Kirk and Hudis, 2008; Pellegrini et al., 2010; Liu et al., 2013), depression (Stanton et al., 2014), and lack of urgency in the absence of cancer symptoms as significant factors for early discontinuation (Jimmy and Jose, 2011). However, other high-quality studies have found no effects for educational level (Wigertz et al., 2012), age, depression (Cluze et al., 2012), tumor stage (Partridge et al., 2010), cancer worry (Brier et al., 2017), or race (Sheppard et al., 2013).

Hobfoll’s Conservation of Resource theory posits that an individual will continually weigh the cost of dealing with a stressful challenge against the resources and capacities in possession. If the cost outweighs the capacity, then the inherent motivation to conserve resources will motivate the individual to reduce the cost (Hobfoll, 1989). To be persistent to ET for 5 years or more, patients must constantly adapt to various challenges within changing contexts over time. Yet nearly all of the aforementioned studies relied on classical regression analyses predicating baseline factors against adherence outcomes that did not occur until months or years later. These models do not account for the changing impact of predictive factors over time, nor do they allow for modeling of decision uncertainty, i.e., the dispersion of outcome probabilities in individuals with identical baseline predictor profiles (Griffin et al., 2006). This may partly explain why the literature on predictive risk factors for early discontinuation is somewhat inconsistent. Variability between individuals with identical characteristics cannot be reduced through measurement but can be characterized with empirical distributions. Complex models are needed that account for the multiple sequential decisions that lead to the end state of remaining adherent or discontinuing ET in context-specific and time-dependent ways.

Recent work by our group describing changes in the concerns of ET patients over the duration of treatment further emphasizes the importance of this time dependency in particular in prolonged treatments such as this that span several years (Shinn et al., 2019). For example, this previous analysis suggested that while worry about bone loss as well as bone and joint pain were consistent concerns contributing to ET discontinuation regardless of treatment phase, concern over diminished sex drive and loss of cognitive function only became significant in mid and late phases, respectively.

The purpose of the present work is to explore this temporality further and gain additional insight into the decisional mechanisms that govern the complex interdependence of patient-, disease-, and patient-provider level factors and their changing roles in prompting discontinuation from ET. To do this, we have adapted a framework originally proposed by Thomas (1991) to study the regulatory dynamics of biological networks. We use the latter to construct a behavioral feedback network that represents our current understanding of the interdependent factors and the cascade of dynamic processes that affect adherence behavior. We then refined the network to reproduce observed behaviors and predict potential intervention strategies directed at deterring discontinuation and restoring adherence.

## Materials and Methods

### Eligibility and Design

Following Institutional Review Board approval at MD Anderson Cancer Center (MDA), patients were consented and enrolled onto study between January 2014 and September 2017. Patients were approached for recruitment during their 6-month surveillance visit at MDA. Patients were eligible if they had been previously treated for stage I–III-hormone receptor positive (HR+) breast cancer, and were ≥18 years of age. Patients were ineligible if they had recurrent disease or a new primary cancer, or could not read English or Spanish well enough to complete the survey.

After enrolling onto the study, participants completed a baseline semi-structured interview by telephone and a baseline questionnaire. Participants completed annual follow-up questionnaires and telephone interviews that also assessed ET adherence. The annual telephone interview took 30–45 min to complete and the questionnaire, 15–30 min. Participants had the option to complete the questionnaire battery online *via* REDCap, in clinic or by mail. Interviewers were trained individually using interview guides, observing live interviews and receiving formal feedback on audiotaped telephone interviews. All interviews were audio-recorded, transcribed and coded by two independent coders. Participants were followed at 6-month intervals for up to 7 years.

### Measures

#### Primary Outcome

Early discontinuation was defined as the participant independently deciding to stop daily ET prior to end of the intended treatment period. From the initial positive reports of the MA.17 trial in 2007 (Goss, 2007) to the 2016 JCO publication showing a 34% reduced risk for recurrence in women who extended ET after completing 5 years of aromatase inhibitors (Goss et al., 2016), the standard of care had clearly shifted toward extension of ET beyond 5 years (Abraham et al., 2016). During the course of our study, all participants in our study were asked to extend ET beyond 5 years, in line with ASCO clinical practice guidelines (Burstein et al., 2019). Early discontinuation was not counted if: (1) the healthcare provider stopped ET, (2) the participant intended to restart the pill at a later date (i.e., self-imposed drug holiday), or (3) the participant was in the midst of transitioning to a different type of ET. Questionnaire and interviews were conducted on an annual basis; discontinuation status and date of discontinuation were assessed by telephone interview every 6 months.

#### Dependent Variables

Selection of patient-level and disease/treatment factors for annual prospective measurement were based on literature review at the initiation of the study, with preference for prospective studies with clearly defined patient characteristics using validated psychometric scales.

##### Patient Factors as Measured With Annual Self-Report Questionnaire

Race, ethnicity, level of education, household income, marital status, occupational status, refill routines, and ET costs were assessed by self-report questionnaire at baseline. Household income, marital status, occupational status, refill routine, and costs were re-assessed at annual follow-ups. Cancer Recurrence Worry was measured with a 4-item, 4-point Likert scale adapted from Lerman’s ovarian Cancer Worry scale (coefficient α = 0.70) asking about level and frequency of worry about recurrence and its impact on mood and daily activities (Lerman et al., 1994). Risk Perception of Cancer Recurrence was assessed with two items asking patients for numerical estimates of personal risk and other breast cancer survivors’ risk and two qualitative items assessing recurrence risk using a 5-point Likert and a binary scale (Lerman et al., 1994; Kelly et al., 2004, 2005). The Numeracy scale assesses capacity for understanding numerical information about risk with a content-neutral 3-item performance test concerning probabilities, percentages, and frequencies (Schwartz et al., 1997; Reyna and Brainerd, 2007). Health literacy was screened with Chew et al.’s (2004) Health Literacy Screening Questions, a 3-item, 5-point Likert scale that assess understanding medical information, confidence in filling out medical forms, and the need for help with reading hospital materials. The screener performed well compared to the Short Test of Functional Health Literacy in Adults (Chew et al., 2004). Treatment Cost Worry was assessed with a single item from McHorney’s 3-item, 5-point Likert Brief Adherence Estimator scale (McHorney, 2009; McHorney et al., 2009) “I feel financially burdened by my out-of-pocket expenses for my hormone treatment pill.” Quality of life (QoL) was measured with the Functional Assessment of Cancer Therapy-General (FACT-G), which measures five domains of QoL: physical function, social/family support, emotional distress, and ability to live a fulfilling life. Overall, the FACT-G has good test-retest (*r* = 0.81) and internal reliability (Cronbach’s α = 0.92), and has demonstrated good convergent, divergent, and criterion validity (Cella et al., 1993). General anxiety unrelated to cancer-related worry was measured with a single, 5-point Likert item from the FACT, “I feel nervous.”

##### Patient Factors Measured With Annual Interview

For behavioral routines, participants were asked *via* semi-structured interview about pill storage, degree of automation in obtaining refills, environmental cues for pill-taking and time of day. Responses were coded, binarized for presence or absence and summed to obtain an index score. To calculate coping deficit, participants were asked during the semi-structured interview if they experienced any of ten individual ET side effects. For each positively endorsed side effect, participants were then asked whether they had tried any coping strategies. For each side effect, a coping deficit score was binarized as either present or absent. All possible values were then summed to obtain a total continuous score ranging from 0 to 10.

Patient-Provider Relationship factors were measured two ways. Patient satisfaction with the healthcare provider was assessed by semi-structured interview focusing on communication about the rationale for ET and patient perception of their provider’s interest and competence in managing ET side effects. Patients also completed the self-report Trust in Physician Scale, an 11-item, 5-point Likert scale measuring the patient’s belief in the doctor’s competence, caring, and integrity (Thom et al., 1999). The scale has demonstrated strong reliability (α ranged from 0.85 to 0.90) and validity.

##### Treatment and Comorbid Factors

Endocrine treatment (ET) Side effects were measured annually by self-report questionnaire using the Breast Cancer Prevention Trial (BCPT) Checklist, which was validated in 2208 breast cancer patients (coeff. α = 0.81) (Stanton et al., 2005) Tumor stage, chemotherapy (yes/no), radiation (yes/no), and surgery (mastectomy vs. lumpectomy) were abstracted from the medical record. Comorbidity was assessed during the annual follow-up interviews with the Charlson Comorbidities checklist at baseline and annual follow-up. It has high test-retest reliability (*r* = 0.91) and good agreement with abstracted medical record review (overall κ = 0.70) (Katz et al., 1996). In addition, annual follow-up questions about non-cancer related illnesses requiring hospitalization were assessed by semi-structured interview.

### Analysis

#### Interview Data Coding and Qualitative Analysis

As a first step in characterizing the development of discontinuation trajectories, qualitative analyses were conducted for each patient’s set of sequential annual interviews to discover key sequences leading to either adherence or discontinuation. Qualitative analysis provides a unique methodology to understand dynamic relationships between factors and provides insight into the patients’ individual perspectives (Sullivan and Sargeant, 2011). Transcripts were independently coded by multiple investigators (NJ, BB, ES) with *a priori* codes from the literature and inductively generated codes, using Atlas.ti8 Windows software (Scientific Software Development GmbH, Berlin, Germany). Disagreements between researchers were used to refine codes and come to agreement about themes and further coding. In order to compare differences in code patterns between adherent and discontinued participants, all coded responses were binarized (code present = 1/code absent = 0) across all timepoints to compare the proportion of adherent vs. discontinued participants who exhibited specific codes. Differences in code occurrences were then further examined to identify emerging themes distinguishing adherent from discontinued patients. To preserve the validity of comparing codes from patients who remained adherent with discontinued patients, only the interviews prior to the discontinuation event were coded and analyzed. Resulting observations of the dynamic interaction between individual demographic and psychosocial factors, patient-provider relationship factors, and ET side effects formed the basis of a behavioral feedback structure capturing the logical paths of sequential decision-making as patients progress through sequential years of adjuvant treatment.

#### A Behavioral Regulatory Model

##### Characteristic Treatment Courses Involving Discontinuation

As our primary objective was to model the occurrence of discontinuation at various points over time during ET (as opposed to persistence with ET), we categorized treatment course or trajectories leading to discontinuation into four characteristic subtypes: (1) initial adherence leading to early and persistent discontinuation, (2) initial adherence leading to mid-course persistent discontinuation, (3) late persistent discontinuation, and (4) transient discontinuation with subsequent recovery of adherence. For each of these characteristic discontinuation trajectories, two participants exhibiting this characteristic behavior were selected with maximally contrasting demographic profiles (age, income, and education), resulting in eight adherence trajectories serving as the representative subset to which the adherence behavior predicted by any potential set of decisional logic parameters would be compared. Alignment error between the predicted adherence behavior and these eight characteristic trajectories was computed as the Manhattan distance separating observed and predicted states at each time point. This overall error was decomposed into deviations at the level of individual nodes and individual observations.

##### A Discrete Decisional Logic

**FIGURE 1:**
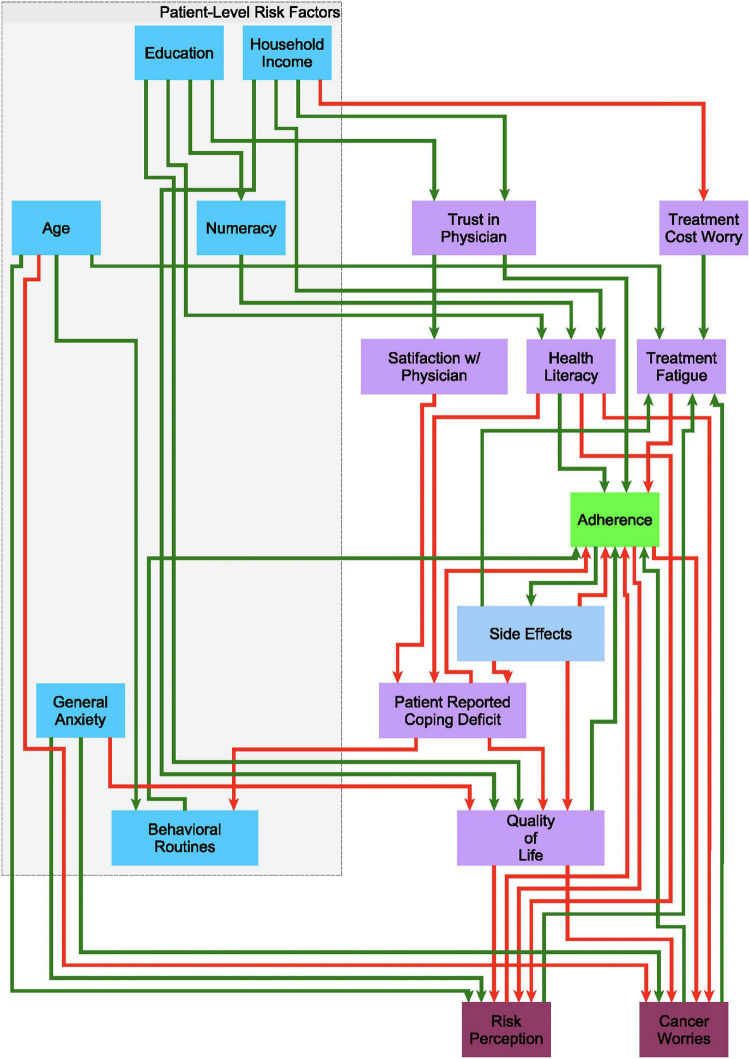
A putative causal behavioral logic circuit supporting adherence to endocrine treatment (ET). A circuit model connecting 18 behavioral and demographic factors through 43 causal regulatory interactions describing the promotion (green arrows) or inhibition (red arrows) of one factor by another in the decision to alter adherence to ET as posited based on clinical experience.

Building on earlier work by Mendoza and Xenarios (2006), we used a discrete decisional logic to describe information flow through a behavioral feedback network where 18 demographic, treatment, and patient-related factors (network nodes) mediate one another dynamically through 43 putative regulatory interactions (network edges) to represent the overall state of the patient’s adherence (Figure 1). Each network edge, or regulatory interaction has a direction, i.e., a source and a target, as well as a mode of action, i.e., inactivating or activating a downstream target. The extent to which each behavioral and demographic node is activated or expressed is described as a discrete qualitative state. Our group has extended the standard two-state Boolean (on-off) description to accommodate additional discrete states of activation, e.g., very low, low, nominal, high, very high, etc… (Sedghamiz et al., 2018). An increase or decrease in the activation level of a node is determined by the states of its upstream neighbors. The competing actions of upstream neighbors expressed above their respective perception thresholds are managed by a decision logic that weighs the actions of weak inactivators against strong activators, and vice versa, in a context-specific way before deciding to increase or decrease the activation of a node in the following iteration (Sedghamiz et al., 2018; see Supplementary Figure 1). Importantly, time is not represented explicitly but instead it is captured implicitly as sequential changes in the evolving pattern of active network nodes. These sets of decisional kinetic parameters (logic weights and perception thresholds) are identified exhaustively by formulating this search as a computationally efficient constraint satisfaction problem (CSP) (Barták, 1999) where combinations of decisional logic parameters are retained if they support predicted dynamic responses that include behaviors observed experimentally (Sedghamiz et al., 2017, 2019a), specifically in this work the recovery of the 8 characteristic treatment trajectories described in Section “Characteristic Treatment Courses Involving Discontinuation.” As more different unobserved events and behaviors may have occurred between longitudinal assessments points there often exist many combinations of these parameters that support the recovery of observed data equally well.

##### Simulation of Transient and Persistent Behaviors

To determine the number and types of stable, persistent behaviors, and corresponding assessment profiles that are supported by the proposed regulatory network (Figure 1), a simulation analysis was conducted. At any given timepoint, each network node was examined separately for its upstream mediators that are expressed above their perception or activation threshold. As described above, based on the specific combination of active upstream mediators and their mode of action (activator or inactivator), the state of the downstream node either remained unchanged, increased or decreased in the next time step. The suggested next state for all nodes constituted the network image, that is the overall state toward which the network should progress given its current state. According to the update scheme being applied, this suggested change in state may be applied to a random node (asynchronous update) or to all eligible nodes simultaneously (synchronous update). As we were primarily interested in stable persistent behaviors, simulations were conducted here using a synchronous update of all node states for reasons of computational efficiency.

We conducted 1,000 repeated simulations where all demographic, treatment, and psychological nodes were set to random combinations of low, nominal, or high activation levels and the network was allowed to migrate toward a stable end-state over 50 iterations. Assigning random combinations of activation levels as the network’s initial departure state in these simulations was done to account for decisional uncertainty as well as mimic unexpected and difficult to capture events that are present in real-life adherence trajectories. The frequency of occurrence of each end-state was recorded and used to provide an estimate of the behavioral inertia to either adherence or discontinuation.

Identifying intervention targets. In much the same way as the above-mentioned strategy for identifying acceptable sets of logic rules, each model can be used to simulate outcomes if specific interventions are applied to targetable psychological nodes at specific points in time. We tested the following changeable targets for adherence: Trust in Physician, Treatment Cost Worry, Health Literacy, QoL, Patient-Reported Coping Deficit, General Anxiety, Worry about Cancer Recurrence, and Behavioral Routines. We then applied a variant of CSP optimization, Answer Set Programming, refined by our group (Guziolowski et al., 2013) to conduct a simulation-based search for minimal intervention sets (MIS). MIS are the most compact combinations of targetable behavioral mediators that if modulated simultaneously or in sequence could disrupt persistent non-adherence in favor of resuming ET. These intervention strategies are also ranked according to the expected promptness of response and robustness to noise (Sedghamiz et al., 2019b).

The computational framework and constituent toolbox components used to identify network model parameters, simulate network response dynamics, and identify optimal intervention strategies were developed by our group using Python version 3.8.3 (2020-05-13).^1^

## Results

### Observed Changes in Adherence Behavior

We enrolled 129 patients who had been diagnosed with HR+ stage I–III breast cancer between 2013 and 2016. During follow-up, 6 participants died, 9 had recurrent cancer, and 32 participants completed fewer than 3 annual assessments; resulting in 82 participants with 3–7 years of annual assessment data. Demographic characteristics of the study population are presented in Table 1. Twenty participants discontinued ET and an additional 5 participants entered the study having already discontinued ET and remained discontinued. The remaining participants (*n* = 57) were adherent upon study entry and remained adherent throughout follow-up.

**TABLE 1:** Study population.

	Discontinued (*n* = 40)		Still taking (*n* = 176)		
Characteristic	*N*	%	*N*	%	*P*-value
Age at study entry					0.623
N	22		60		
Mean (SD)	64.0 (13.24)		63 (12.03)		
Median	64.1		63.5		
Min-Max	40–88		36–91		
Race					0.987
White	20	90.9	47	78.3	
African American	2	9.1	6	10.0	
Asian	0	0.0	4	6.7	
Other	2	9.1	3	5.0	
Ethnic background					0.393
Hispanic	12	54.5	39	65.0	
Not Hispanic	10	45.5	21	35.0	
Education level					0.874
High school or GED	4	18.2	10	16.7	
Some college	8	36.4	12	20.0	
College degree	8	36.4	22	36.7	
Master’s or higher	5	22.7	16	26.7	
Marital status					0.007
Married	20	90.9	39	65.0	
Never married	1	4.5	4	6.7	
Divorced	0	0.0	11	18.3	
Widowed	1	4.5	6	10.0	
Breast cancer stage					0.980
Stage I	6	27.3	17	28.3	
Stage II	11	50.0	29	48.3	
Stage III	5	22.7	14	23.3	
Surgery					0.934
Mastectomy	21	95.5	57	97.0	
Lumpectomy	1	4.5	3	5.0	
Chemotherapy					0.232
No	19	86.4	45	75.0	
Yes	3	13.6	15	25.0	
Radiation					0.566
No	10	54.5	23	61.7	
Yes	12	45.5	37	38.3	
Number of assessment timepoints					0.081
Mean (SD)	4.5 (1.14)		4.1 (1.01)		
3	5	22.7	22	36.7	
4–7	17	77.3	38	63.3	

*Demographic and clinical characteristics of participants with 3–7 years annual data (n = 82).*

During the course of the study, there were 22 documented changes in adherence status. Twenty participants moved from adherent to discontinued status, whereas two participants who had discontinued reverted back to adherence during later years (Supplementary Table 1). Seven of the discontinuation events occurred before the end of the fifth year and an additional 13 discontinued during the sixth, seventh, and eighth years of ET.

Code co-occurrence analysis of the coded transcripts uncovered several observations that formed the basis of the decisional network design characterizing an individual’s propensity to either remain adherent to ET or to discontinue. Compared to adherent patients, participants who discontinued had similar levels of side effects, but were more likely to perceive significant decreases in QoL and downplay the risk of cancer recurrence. The impact of side effects on QoL was mediated by perceived helpfulness of the healthcare team as well as age. Higher age, lower education, lower disease stage, and poor understanding of the rationale for ET was related to lower perceived risk of cancer recurrence. Discontinued participants were also more likely to exhibit inconsistencies with pill-taking routines compared to adherent participants and were more likely to report coping deficits with the various challenges associated with maintaining adherence.

We used these observations and findings from the literature (regarding demographics, health literacy, and numeracy, among other constructs) to form the initial putative structure and flow of information within the network model of adherence over time (Figure 1).

### Mechanistic Hypotheses of Discontinuation

The search for model parameters yielded 96 candidate adjustments to the decisional logic dictating the responses available to the regulatory circuit model in Figure 1. Recall that in each case, given the current adherence status, expression of beliefs, concerns and other psychometric descriptors of behavior, the network circuit will apply a set of these decisional logic parameters to compute the next expected behavioral profile and adherence status. Note that the decisional dynamics are represented in the model as one-step forward prediction and that the notion of absolute time (e.g., year 1, 2, etc…) is not explicitly described but rather implicitly captured by the changing behavioral profile and adherence status. We propose that this is more generalizable as an individual may experience a level of treatment fatigue in early course that another might only experience much later, both of which might motivate discontinuation regardless of the absolute duration of ET. Each of 96 competing sets of decisional logic rules identified here, when applied to regulatory circuit in Figure 1, captured up to 75% of the coordinated changes in the 16 network nodes across time. The Manhattan distance between the predicted and observed states suggested that comparable accuracy was obtained for each of the eight paths to discontinuation; no single behavioral trajectory was more poorly represented than the other (Supplementary Figure 2A). In contrast to this, an analysis of the error in predicting the expression of each individual network node across time showed that changes in worry of cancer recurrence (5%), general anxiety (2.9%), and trust in physician (2.9%) had the highest error percentages among the nodes (Supplementary Figure 2B).

### Stable Persistent Behaviors

As mentioned in the previous section, time is represented in relative terms and is described by changes in behavioral context, which might occur sooner in some and later in others. Independently of absolute duration of ET, circumstances might promote changes in concerns, beliefs and behaviors that become established. In some cases, these will lead to persistent discontinuation that is galvanized by a self-reinforcing behavioral feedback having become active. In such a regulatory trap, given my current behavioral state, I will continue to feel the same way, believe the same things and decide to maintain my current adherence status in the next time step. Simulations predicting the sequence of decisions supported by the regulatory network, given different initial psychometric and demographic profiles, can be used to characterize conditions that favor the onset of such persistent behaviors and the specific combinations of concerns and beliefs that render them so resistant to change. Conducting such simulations with each of the 96 candidate models over a broad range of initial demographic and behavioral profiles, we identified a total of 15 stable behavioral traps, all of which led to persistent discontinuation (i.e., none of the 15 persistent states described naturally occurring processes resulting in a state of stable self-perpetuating adherence). These 15 self-perpetuating profiles were uniformly characterized by low risk perception, below-median QoL, low ET-related side effects, low level of cancer recurrence worry, and median levels of general anxiety. Education, trust in physician, health literacy, and age did not exhibit consistent effects (Supplementary Figure 3). Of these 15 natural resting states, 9 were unanimously supported by all 96 models.

### Candidate Intervention Strategies

Recall that behavioral feedback described by the network model actively perpetuates each of these 15 states of discontinuation making the latter especially refractory to change. As a result, we expect that breaking out of such behaviors will require targeted intervention that in some cases involves more than one modality. To identify the simplest and most effective intervention strategies that might be effective in specific demographic sub-populations, we apply a simulation-based search for MIS, or network targets that if modulated in a specific way, either alone or in combination, would disrupt a self-reinforcing state of non-adherence in favor of a return to adherence. Conducting such simulations with all 96 model variants, we found MIS that supported a sustained transition to adherence from 13 of the 15 non-adherent steady states by selectively targeting one or two clinically actionable behavioral mediators. All 96 candidate models unanimously supported the use of three distinct intervention strategies: reinforcing behavioral routines, improving trust in the physician, and reducing levels of general anxiety. Reinforcing behavioral routines or trust in physician both disrupted persistent discontinuation and promoted a return back to adherence in all 13 treatable non-adherent psychometric profiles.

**FIGURE 2:**
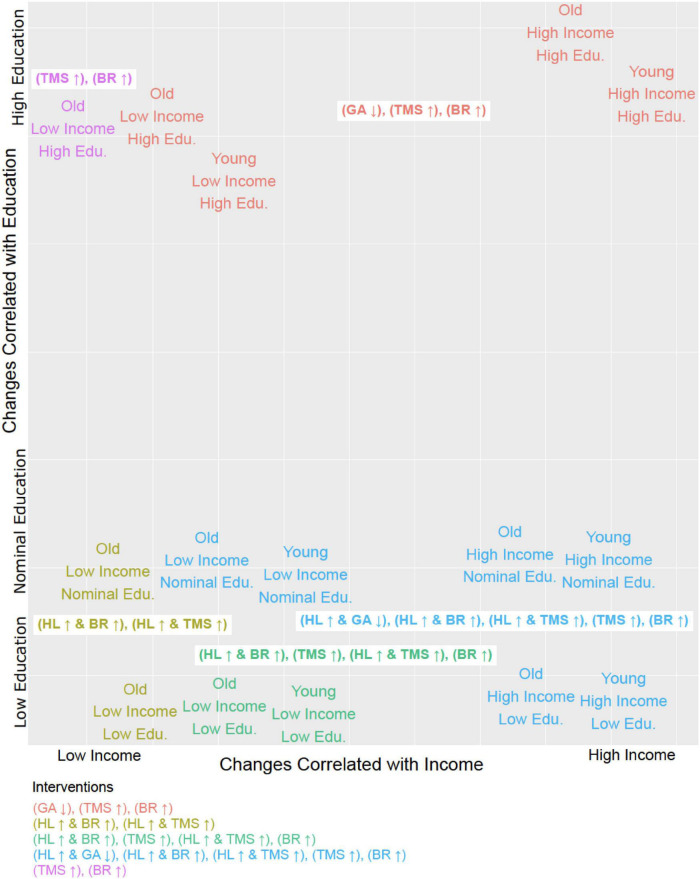
Tailoring interventions to demographic profiles. Each of the 15 steady states identified in terms of its corresponding demographic profile (i.e., Young, High Income, Low Education would be Age = 0, Household Income = 0, Education = 0) plotted in relation to the six distinct intervention strategies (MIS, minimal intervention sets) and it’s effective in treating that group. The MIS abbreviations are as follows: General Anxiety (GA), Health Literacy (HL), Beneficial Behavioral Routine (BR), and Trust in Physician (TMS). The arrow next to the abbreviation indicates whether the entity is up or downregulated. All intervention strategies described by these MIS suffice to restore and maintain adherence.

The suitability of these focused single-factor intervention strategies for subgroups of a specific age, income, and educational profile is illustrated in Figure 2. Combining a fourth intervention strategy, improving health literacy, with each of the three aforementioned strategies yielded a higher number of sustained transitions back to adherence for patients who were older (aged 56 or more), lower-income (annual household income of $50,000 or less), or without a college degree (Table 2). Reducing anxiety level was successful in four of the 13 profiles but did not result in sustained transitions back to adherence for participants without a 4-year college degree, regardless of income or age.

**TABLE 2:** Idealized intervention strategies.

	Persistent non-adherent state demographic characteristics							
	Age		Annual income		Education			
	Younger (38–59 years)	Older (60–100 years)	Lower (<$50 k/yr)	Higher (>$50 k/yr)	Low (High school or less)	Nominal (Some college/tech. school)	High (4-year college or more)	Total non-adherent steady states that can be rescued
**Single point intervention**	**# of non-adherent states that can be rescued**							
↑ Beneficial routine	6	7	7	6	4	4	5	**13**
↑ Trust in medical system	6	7	7	6	4	4	5	**13**
↓ Generalized anxiety	2	2	2	2	0	0	4	**4**
**Combination intervention^1^**								
↑ Health literacy + ↑ Beneficial routine	4	6	6	4	5	5	0	**10**
↑ Health literacy + ↑ Trust in medical system	4	6	6	4	5	5	0	**10**
↑ Health literacy + ↓ Generalized anxiety	3	3	2	4	2	4	0	**6**

*Single and multi-factorial intervention strategies predicted to disrupt persistent non-adherent behaviors in favor of recovering continued adherence to ET.*

*^1^Concurrently increasing health literacy in patients with lower education levels was predicted to improve efficacy of the aforementioned strategies.*

## Discussion

There were several key findings in our study. First, comparison of codes generated from adherent versus discontinued patients revealed that the levels of side effects in adherent patients were not lower compared to the levels found in patients before they eventually discontinued. Using Hobfoll’s Conservation of Resource theory to interpret our analytic results from AtlasTi8, we found that all participants viewed the decision to maintain adherence or not by weighing the impact of ET side effects against QoL. This finding is consistent with other studies which identified cognitions about the experience of bone and joint pain as a key predictor of non-adherence to ET, rather than the level or presence of musculoskeletal pain (Brier et al., 2017). While QoL was a key factor in patients’ adherence decisions, our qualitative analysis also revealed that high fear of cancer recurrence outweighed the importance of unacceptable decreases in QoL.

Using Hobfoll’s theoretical framework as a guide, we created a behavioral feedback model which incorporated dynamics derived from both the qualitative analysis and from the extant literature predicating patient demographics, psychosocial attributes, patient-physician relationship, and treatment factors with adherence to adjuvant ET. Selection of patient-level and disease/treatment factors were based on literature review, with preference for prospective studies with clearly defined patient characteristics using validated psychometric scales. Within the body of ET adherence literature that included *a priori* measurement of psychosocial factors, moderate but inconsistent effects have been found for depression (Bender et al., 2014), anxiety (Stanton et al., 2005), and social support (Hurtado-de-Mendoza et al., 2016). More consistent patient-level factors include number of comorbidities, poor physician-patient relationship (Liu et al., 2013), and side effect severity (Stanton et al., 2005; Cluze et al., 2012). The initial behavioral feedback network captured the logical path of sequential decision-making leading toward eventual discontinuation or adherence as patients progress through multiple years of adjuvant treatment.

We further refined the structure of this original network model by applying a discrete decisional logic to direct the flow of information through the behavioral feedback network and predict how the component constructs evolve across time (Sedghamiz et al., 2019a). Toward this we adapted a formalism describing regulatory dynamics in biological networks originally proposed by Thomas (1991), where cascading dynamic responses within a circuit of directed interactions will favor an eventual return to one or several homeostatic resting states. Predicted behaviors were compared to quantitative measurement of these constructs to determine the logic models that best reflected characteristic patient trajectories leading either to discontinuation or adherence over time. The resulting knowledge was applied to the simulation and design of potential intervention strategies which would reverse discontinuation trajectories (Sedghamiz et al., 2019b).

A main finding of simulations conducted across the family of 96-candidate adherence logic networks was the emergence of 15 self-perpetuating and dynamically stable behaviors, all of which galvanize non-adherence. In other words, adherence to ET is not a spontaneous or naturally occurring behavior. Simulating adherence behavior change across time in various combinations of patient-, treatment-, and patient-provider factors tended to result in an eventual discontinuation end-state. This finding may explain breast cancer patients’ high rates of early discontinuation from ET and the continuous effort that patients must exert in order to remain adherent for several years. That our model captured 75% of the coordinated changes in the 16 demographic and behavioral network nodes over time suggests that qualitative analysis was useful in elucidating dynamic relationships between multiple factors. To our knowledge, this is the first attempt to combine qualitative data analysis with computationally efficient methods of characterizing and simulating the regulatory dynamics that emerge from interactions between demographic, psychological, and treatment-related variables in patient adherence pathways over time. Early, mid-, late-, and transient discontinuation pathways were explained equally well by the 96 candidate models, suggesting that the effects of treatment duration can be better represented in subject-specific relative time that is implicitly expressed through changes in concerns and beliefs currently experienced by an individual.

Encouragingly model predictions suggest that 13 of the 15 states of stable persistent non-adherence could be theoretically rescued by targeting three psychological factors: behavioral routines, trust in physician, and general anxiety. In particular, improving patient trust in the physician and behavioral reinforcement of daily pill-taking routines may be especially promising, as these two intervention targets reversed 13 discontinued states across a broad range of demographics. Existing medication adherence intervention research has tended to focus on scalable but limited strategies, such as changes in medication packaging, reminder texts or educational newsletters. As such, very few randomized control trials of adherence interventions have shown sustained improvement (Markopoulos et al., 2011; Hadji et al., 2013a; Schneider et al., 2014; Arthurs et al., 2015; He et al., 2018). A multi-site trial of 724 breast cancer patients which randomized patients to receive brief text messages twice a week for 3 years versus no texts showed no significant differences between groups (Hershman et al., 2020). Other adherence intervention trials have mailed educational newsletters about ET but these too were found to be non-efficacious (Markopoulos et al., 2011; Yu et al., 2012; Hadji et al., 2013a,b; Hershman et al., 2020). Derived from qualitative data analysis, our definition of reinforcing behavioral routines included improving consistency in the timing of daily pill-taking, automation of various aspects of refill procedures, and using environmental cues. In support of our finding that increasing patient trust in the physician may improve adherence, two studies which utilized two-way communication between the patient and oncology treatment team demonstrated significant increases in ET adherence (Ziller et al., 2013; Graetz et al., 2018). Future intervention work may benefit from emphasizing these two strategies in addition to traditional targets in psychosocial programs.

While a substantial proportion of decisions were captured by the model, no model is complete. Our analysis of contributions by each of the 16 network nodes to these decisions suggests future work should focus on the inclusion of additional regulatory processes as opposed to increasing the variety of discontinuation trajectories. Indeed, these preliminary models found that regulation of worry about cancer recurrence by the behavioral feedback network deviated from observed patterns by almost 5% error. Therefore, the location of this measure in the behavioral network and its regulatory relationships would benefit from additional study. The inclusion of new network nodes representing other factors also promises to further improve the accuracy, for example, nodes describing personality traits and the influence of psychological disorders. While we expect this to be true, the selection of new measures should be approached with caution. Because reports regarding these personality traits and psychopathologies such as generalized anxiety and depressive disorders have been inconsistent, and because our qualitative analysis did not uncover these codes frequently within patients’ paths to discontinuation, we chose to constrain network behavior to reproduce changes in those patient-level factors that had either consistency within the extant literature (e.g., number of comorbidities, poor physician-patient relationship) or were indicated as contributing toward the tendency of eventual discontinuation by qualitative analysis. However, it should be pointed out that within the clinical trial literature, examination of patient-level factors is limited to those measures that can easily be abstracted from clinical study records or, in the case of large retrospective studies, the medical record. Furthermore, patients with documented psychiatric disorders are often ineligible from clinical trials. Therefore, our initial factor selection for the prospective study may have been biased against including psychosocial factors.

Though this network informed approach to describing adherence behavior shows promise, we must also caution that generalizability of our findings was limited by our sample being racially homogeneous and from a single institution. Also, because we only included participants with at least three annual years of data, our sample may have been biased to be more adherent than that found in the general ER+ breast cancer patient population. Given the propensity of our sample to remain adherent, it is interesting that none of the predicted change states gravitated to adherence. Finally, given the relatively small number of changes in adherence status in our sample, some of our findings may be spurious. These limitations notwithstanding, we believe that this is a novel way to model inductively generated knowledge about the complex dynamics of multifactorial and context-specific behavior in a testable manner.

## Data Availability Statement

The raw data supporting the conclusions of this article will be made available by the authors, without undue reservation.

## Ethics Statement

The studies involving human participants were reviewed and approved by IRB-4, MD Anderson Cancer Center. The patients/participants provided their written informed consent to participate in this study.

## Author Contributions

ES and WS conceived and designed the study. GB and JT conceived and designed the network simulation analyses. ES, BB, and NJ collected the data and coded the interviews. ES, BB, and SP analyzed the qualitative interviews in AtlasTi8. MC-M and WS provided clinical interpretation of analytic results. JT, SR, and CL discretized the data, designed and tested the network model, and conducted all parameter identification, simulation, and optimal intervention analyses. All authors were involved in manuscript writing and approval of the manuscript.

## Conflict of Interest

The authors declare that the research was conducted in the absence of any commercial or financial relationships that could be construed as a potential conflict of interest.

## Publisher’s Note

All claims expressed in this article are solely those of the authors and do not necessarily represent those of their affiliated organizations, or those of the publisher, the editors and the reviewers. Any product that may be evaluated in this article, or claim that may be made by its manufacturer, is not guaranteed or endorsed by the publisher.

## Footnotes

1
https://www.python.org/
