# Supplementary material for: Network Modeling of Complex Time-Dependent Changes in Patient Adherence to Adjuvant Endocrine Treatment in ER+ Breast Cancer
Source: Front Psychol. 2022 Jul 12;13:856813. doi: 10.3389/fpsyg.2022.856813 (PMC9315289; doi:10.3389/fpsyg.2022.856813)
Supplement: Supplementary file 1 [file Data_Sheet_1.pdf]

# *Supplementary Material*

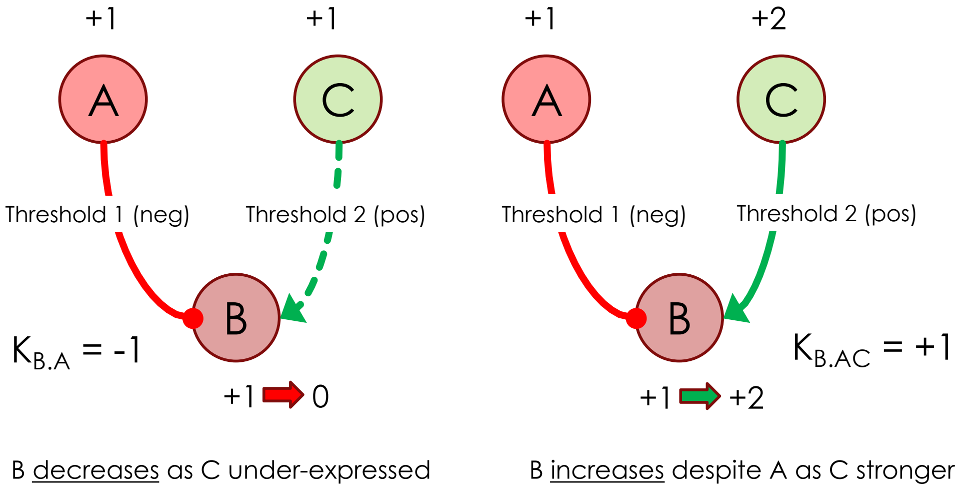


**Supplementary Figure 1.** *Discrete logic decisional parameters*. In a basic circuit a node B can be downregulated by node A and upregulated by node C. In the left panel, when A is expressed at a state +1 in excess of its activation threshold whereas C is expressed at state +1 below its activation threshold, then node B is downregulated by node A acting alone ($K_{B,A}$ = -1). In the right panel, when A is expressed at a state +1 in excess of its activation threshold but C is further expressed at state +2 above its activation threshold, then node B is regulated by both nodes. The net decisional weight of nodes A and C acting on B ($K_{B,AC}$ =+1) is such that the net regulatory action is to upregulate B.

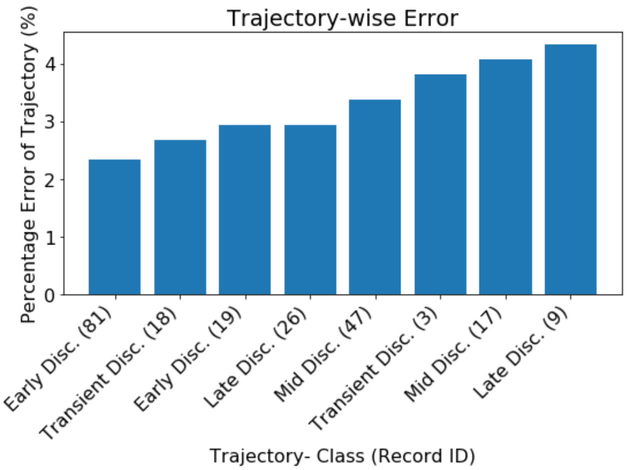


**(A)**

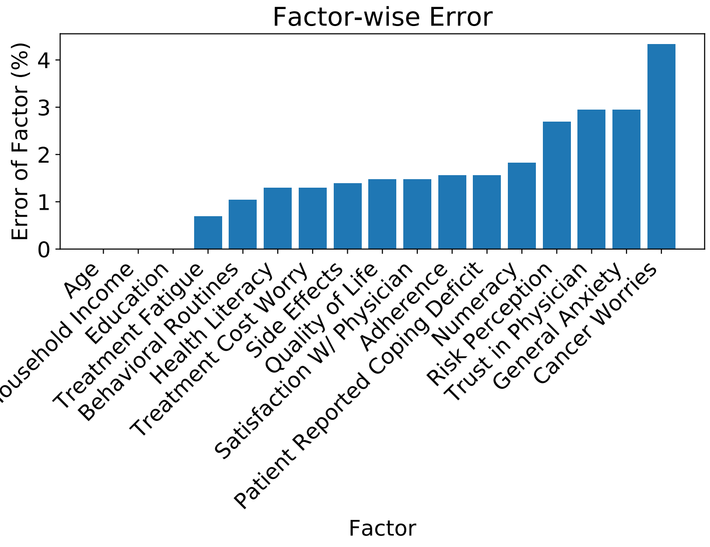


**(B)**

**Supplementary Figure 2.** *Deviation from observed characteristic behaviors and assessment profiles*. A decomposition of the overall deviation in the predicted and measured states as described in terms of all 18 factors at every point in time across all 8 characteristic trajectories (as a % of the maximum possible error) suggests that **(A)** characteristic trajectories are predicted with comparable accuracy and there is no obvious trend with time of discontinuation. We also find that **(B)** the mediation of contextual changes across time for worry of cancer recurrence, generalized anxiety and trust in the medical system are less consistent with the proposed mechanistic model, showing the highest node-wise error, which suggests that regulation of these factors is less well understood.

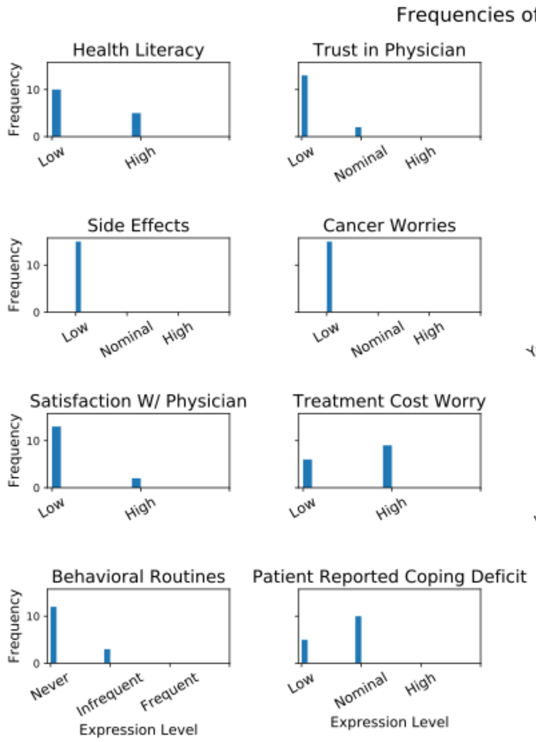


**Supplementary Figure 3.** *Characteristic Self-perpetuating Profiles* The frequency distribution in demographic factors and the expression of individual beliefs, concerns and psychometric measures that are self-perpetuating at 15 stable resting states supported across all 96 competing models.

**Supplementary Table 1.** *Summary of Adherence Decisions.* Documented changes in the adherence status of subjects across 12-month increments.

| Year of ET | Became Adherent | Became Nonadherent |
|---|---|---|
| 1st year | 0 | 2 |
| 2nd yr | 0 | 2 |
| 3rd yr | 0 | 1 |
| 4th yr | 2 | 2 |
| 5th yr | 1 | 7 |
| 6th yr | 0 | 5 |
| 7th yr | 1 | 2 |
| 8th yr | 1 | 0 |
| Total | 5 | 21 |
